# Genetic polymorphisms of 21 STR loci of Golden*e*ye™ DNA ID 22NC kit in five ethnic groups of China

**DOI:** 10.1080/20961790.2018.1479148

**Published:** 2018-11-08

**Authors:** Jiashuo Zhang, Yun Bao, Ruiyang Tao, Fei Guo, Xiang Sheng, Yingnan Bian, Xiling Liu, Suhua Zhang, Chengtao Li

**Affiliations:** Department of Forensic Science, Medical School of Soochow University, Suzhou, China; Shanghai Key Laboratory of Forensic Medicine, Shanghai Forensic Service Platform, Academy of Forensic Sciences, Shanghai, China; Department of Forensic Medicine, Inner Mongolia Medical University, Hohhot, China; Department of Forensic Genetics, West China School of Basic Science and Forensic Medicine, Sichuan University (West China University of Medical Sciences), Chengdu, China; Department of Forensic Medicine, Criminal Investigation Police University of China, Huanggu District, Shenyang, China; Shanghai Key Laboratory of Forensic Medicine, Shanghai Forensic Service Platform, Academy of Forensic Sciences, Shanghai, China zsh-daisy@163.com; Shanghai Key Laboratory of Forensic Medicine, Shanghai Forensic Service Platform, Academy of Forensic Sciences, Shanghai, China lichengtaohla@163.com

Dear Editor, Short tandem repeats (STRs), polymorphic DNA regions with a variable number of repeated units (2–6 base pairs), are attractive to forensic applications such as human identification and parentage testing [1]. Nowadays, most of the commercial STR kits are designed based on STRs from the combined DNA index system (CODIS), European Standard Set (ESS), expanded CODIS, and extended ESS [2]. In this study, we evaluated 21 STRs from Golden*e*ye™ DNA ID 22NC kit (PeopleSpot Inc., Beijing, China), which including 20 polymorphic non-CODIS STR loci (i.e. *D1S1656*, *D2S441*, *D3S1744*, *D3S3045*, *D4S2366*, *D5S2500*, *D6S477*, *D7S1517*, *D7S3048*, *D8S1132*, *D10S1248*, *D10S1435*, *D11S2368*, *D13S325*, *D14S608*, *D15S659*, *D17S1290*, *D18S535*, *D19S253*, *D22GATA198B05*) and a CODIS STR locus (*D3S1358*), in five ethnic groups (i.e. Eastern Han, Ningxia Hui, Xinjiang Uygur, Xizang Tibetan, and Inner Mongolia Mongolian) of China. The forensic genetic investigation of above loci may provide more genetic information in complex kinship testing and population studies [3, 4].

China consists 56 ethnic groups, including the largest Han population and other 55 minority ethnic groups. Here, the five studied ethnic groups were labelled in Figure S1. Blood samples were collected from unrelated individuals of Eastern Han (*n* = 100), Ningxia Hui (*n* = 102), Xinjiang Uygur (*n* = 101), Xizang Tibetan (*n* = 100), and Inner Mongolia Mongolian (*n* = 101) with informed consent. Genomic DNA was extracted with QIAamp DNA Blood Mini kit (Qiagen, Hilden, Germany). The 21 STRs and an Amelogenin locus were co-amplified with Golden*e*ye™ DNA ID 22NC kit on GeneAmp PCR 9700 thermal cycler (Thermo Fisher Scientific, Waltham, MA, USA) according to the manufacturer’s recommendations. Amplified PCR products were detected on the 3130*XL* Genetic Analyzer (Thermo Fisher Scientific). STR profiles were analysed with GeneMapper ID version 3.2.1 software (Thermo Fisher Scientific).

Allele frequencies, Hardy–Weinberg equilibrium (HWE) and forensic parameters, including observed heterozygosity (Ho), expected heterozygosity (He), matching probability (MP), power of discrimination (PD), power of exclusion (PE), typical paternity index (TPI), and polymorphism information content (PIC), were calculated using a modified PowerStats Version 1.2 (Promega, Madison, WI, USA) [5].

To estimate the genetic relationship between the five studied ethnic groups and other ethnic groups, available allele frequencies data of 6914 Han Chinese individuals (Guangxi Han [6], Guangdong Han [7], Hebei Han [8], Hunan Han [9]) and 1 715 minority individuals (Tujia [10], Bai [11], Yi [12], Salar [13], Kazak [14]) were referred. The detailed data source is listed in Table S1. Pairwise genetic distance (fixation index, *F*_st_), also known as Nei’s Gst [15], and analysis of molecular variance (AMOVA, based on *F*-statistics) were performed using the Arlequin v3.5 software [16]. The multidimensional scaling (MDS) plots were generated based on *F*_st_ values between all pairs of ethnic groups using the ALSCAL Multidimensional Scaling Program in IBM SPSS Statistics 23 software (IBM Corp., Armonk, NY, USA). The Neighbour-Joining (NJ) tree was constructed using the “nj” function in the R package “ape” (http://www.r-project.org).

**Table S1:** Detail information of nine referenced ethnic groups

Population	Sample Size	Residence	Reference
Han_Guangxi	152	Guangxi, China	[6]
Han_Guangdong	5234	Guangdong, China	[7]
Han_Hebei	1027	Hebei, China	[8]
Han_Hunan	501	Hunan, China	[9]
Tujia	107	Enshi Tujia Autonomous Prefecture, Hubei, China	[10]
Bai	1158	Dali, China	[11]
Yi	110	Yunnan, China	[12]
Salar	120	Xunhua Salar Autonomous Prefecture, Qinghai, China	[13]
Kazak	114	Ili Kazak Autonomous Prefecture, Xinjiang, China	[14]

Full genotyping profiles were obtained with all tested samples. Allele frequencies and corresponding forensic parameters of the 21 STR loci in the five studied ethnic groups are listed in Tables S^2^–S^6^, respectively. No significant deviation from HWE was observed at the 21 STRs within the five ethnic groups after Bonferroni correction [17] (*P* > 0.05/21), except *D11S2368* in Xizang Tibetan group with *P-*value of 0.0005. We assumed this may be related to the limited sample size. A total of 974 alleles and 778 genotypes were observed, and allele frequencies ranged from 0.0046 to 0.4700. In Xinjiang Uygur, PIC ranged from 0.7015 (*D3S1358*) to 0.8589 (*D7S1517*). For other four studied ethnic groups, PIC ranged from 0.623 to 0.866, with 95% (20 out of 21) over 0.7. Since the 21 STR loci were independent from each other after linkage disequilibrium testing, the combined power of discrimination (CPD) ranged from 1–4.0699 × 10^−24^ (Xizang Tibetan) to 1–1.0280 × 10^−25^ (Xinjiang Uygur), and the combined power of exclusion (CPE) ranged from 1–4.1056 × 10^−9^ (Eastern Han) to 1–2.4912 × 10^−10^ (Xinjiang Uygur). Above results indicated that the 21 STRs are highly polymorphic in the five test ethnic groups, and suitable for human identification and parentage testing.

Table S7 listed the average *F*_st_ values and corresponding *P*-values of compared groups. *F*_st_ values of Guangdong Han-Kazak, Bai-Mongolian, Bai-Hui, Bai-Tibetan, Bai-Uygur, Yi-Hui, Yi-Mongolian, Yi-Tibetan, Yi-Uygur, Salar-Mongolian, Kazak-Mongolian, Kazak-Hui, Kazak-Tibetan, were all above 0.15, showing great genetic differentiation. We also found that Inner Mongolia Mongolian, Ningxia Hui, Xinjiang Uygur, and Xizang Tibetan show moderate genetic differentiation (*F*_st_>0.1) with nine referenced ethnic groups [6–14]. Visual results are shown in Figure S2. The genetic distance among Han populations ranged from 0.0003 to 0.030 8, which was consistent with the results reported by Lu et al. [18]. Among minority ethnic groups, Tibetan and Uygur were found to be the most closely related to Mongolian (*F*_st_=0.0022) and the most distantly related to Bai (*F*_st_=0.1689). We also note that Hui, Mongolian, Tibetan and Uygur were significantly different from Tuijia, Bai, Yi, Salar, and Kazak.

To further improve the visualisation of above results, a two-dimensional MDS plot was constructed (Figure S3). From Figure S3, the following three clusters were observed: Eastern Han cluster closest with Hebei Han, followed by with Guangdong Han and Guangxi Han; Mongolian, Tibetan, Hui, and Uygur shared a closer genetic distance; other ethnic groups clustered together. The above clustering pattern of MDS plot was further supported by NJ tree (Figure S4). From Figure S4, Eastern Han was observed to cluster with Hebei Han, Guangdong Han and Guangxi Han together. Tibetan, Mongolian, Hui and Uygur shared the same clade.

In summary, these 21 STR loci of Golden*e*ye™ DNA ID 22NC kit were highly genetically polymorphic in the five ethnic groups. Thereby, they are effectively valuable for discriminating individuals and testing kinship. Also, the 21 STR loci data obtained in this study could also be useful for growing up STR database for forensic application. In addition, the phylogenetic analysis based on the 21 STRs can reflect the evolutionary relationships, which can increase our understanding of the genetic background.

The paper was written in accordance with the standards for publication of population data [19] and follows International Society for Forensic Genetics (ISFG) recommendations on nomenclature of STR typing systems [20].

Jiashuo Zhang *Department of Forensic Science, Medical School of Soochow
University, Suzhou, China* *Shanghai Key Laboratory of Forensic
Medicine, Shanghai Forensic Service Platform, Academy of Forensic Sciences, Shanghai,
China* Yun Bao *Department of Forensic Medicine, Inner
Mongolia Medical University, Hohhot, China* Ruiyang
Tao *Shanghai Key Laboratory of Forensic Medicine, Shanghai Forensic Service
Platform, Academy of Forensic Sciences, Shanghai, China* *Department
of Forensic Genetics, West China School of Basic Science and Forensic Medicine, Sichuan
University (West China University of Medical Sciences), Chengdu, China*
Fei Guo *Department of Forensic Medicine, Criminal Investigation Police
University of China, Shenyang, China* Xiang
Sheng *Department of Forensic Science, Medical School of Soochow University,
Suzhou, China* Yingnan Bian and Xiling Liu *Shanghai Key
Laboratory of Forensic Medicine, Shanghai Forensic Service Platform, Academy of Forensic
Sciences, Shanghai, China* Suhua Zhang *Shanghai Key
Laboratory of Forensic Medicine,* *Shanghai Forensic Service
Platform, Academy of* *Forensic Sciences, Shanghai,
China* zsh-daisy@163.com Chengtao
Li *Department of Forensic Science, Medical School of Soochow University,
Suzhou, China* *Shanghai Key Laboratory of Forensic Medicine,
Shanghai Forensic Service Platform, Academy of Forensic Sciences, Shanghai,
China* lichengtaohla@163.com
