# Expression of Concern: Genetic Polymorphisms of 21 STR Loci of Goldeneye^TM^ DNA ID 22NC Kit in Five Ethnic Groups of China

**DOI:** 10.1093/fsr/owae044

**Published:** 2024-08-06

In 2018, *Forensic Sciences Research* published the article ``Genetic Polymorphisms of 21 STR Loci of Goldeneye^TM^ DNA ID 22NC Kit in Five Ethnic Groups in China'' by Jiashuo Zhang, Yun Bao, Ruiyang Tao, Fei Guo, Xiang Sheng, Yingnan Bian, Xiling Liu, Suhua Zhang, Chengtao Li: https://doi.org/10.1080/20961790.2018.1479148

In July 2024, the editors and the publisher received concerns regarding a discrepancy between the `Compliance with ethical standards' statement in the article, which indicates that the article does not contain any studies with human participants, and the article itself, which describes the collection of blood samples from human participants. We alert our readers that, pending the outcome of the full investigation, concerns have been raised about this article.

