# Correction to: Genetic Polymorphisms of 21 STR Loci of Goldeneye^TM^ DNA ID 22NC Kit in Five Ethnic Groups of China

**DOI:** 10.1093/fsr/owaf009

**Published:** 2025-06-04

This is a **correction** to:

Jiashuo Zhang, Yun Bao, Ruiyang Tao, Fei Guo, Xiang Sheng, Yingnan Bian, Xiling Liu, Suhua Zhang, Chengtao Li, Genetic Polymorphisms of 21 STR Loci of Goldeneye^TM^ DNA ID 22NC Kit in Five Ethnic Groups of China, *Forensic Sciences Research*, Volume 4, Issue 4, December 2019, Pages 348–350, https://doi.org/10.1080/20961790.2018.1479148

After receiving concerns in July 2024 about the `Compliance with ethical standards' statement in the article, the journal posted an Expression of Concern in September 2024.^1^ The authors subsequently provided the necessary documentation, and the ``Compliance with ethical standards'' statement is hereby corrected to read: ``This study involves human subjects, and the research has been approved by the Ethics Committee of Academy of Forensic Science, Ministry of Justice, China, reference number 2014-W5. Informed consent was obtained from all individual participants included in this study.''

Additionally, Supplementary Figures S1-S4 and Supplementary Tables S2-S7, which were missing in the article, have been added to this notice.

These details have been corrected only in this **correction notice** to preserve the published version of record.

^1^Expression of Concern, *Forensic Sciences Research*, Volume 9, Issue 3, September 2024, https://doi.org/10.1093/fsr/owae044

## Supplementary Material

Figure_S1_owaf009

Figure_S2_owaf009

Figure_S3_owaf009

Figure_S4_owaf009

Table_S1_owaf009

Table_S2_owaf009

Table_S3_owaf009

Table_S4_owaf009

Table_S5_owaf009

Table_S6_owaf009

Table_S7_owaf009

