# Supplementary figures and images for: Correction to: Genetic Polymorphisms of 21 STR Loci of GoldeneyeTM DNA ID 22NC Kit in Five Ethnic Groups of China
Source: Forensic Sci Res. 2025 Jun 4;10(2):owaf009. doi: 10.1093/fsr/owaf009 (PMC12133678; doi:10.1093/fsr/owaf009)

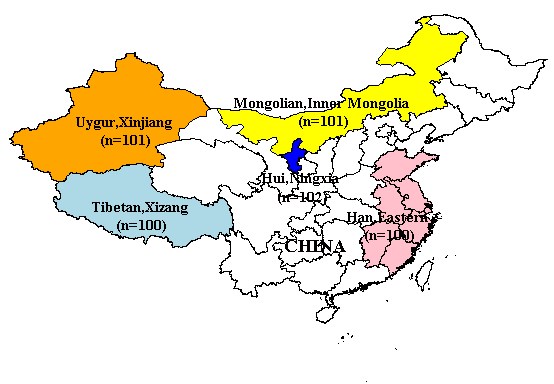

Supplement: Figure_S1_owaf009 [file figure_s1_owaf009.jpeg]

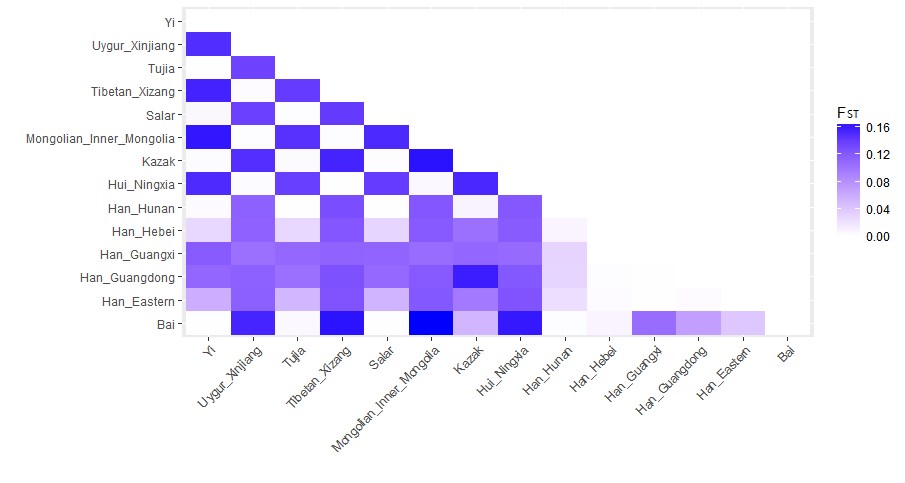

Supplement: Figure_S2_owaf009 [file figure_s2_owaf009.jpeg]

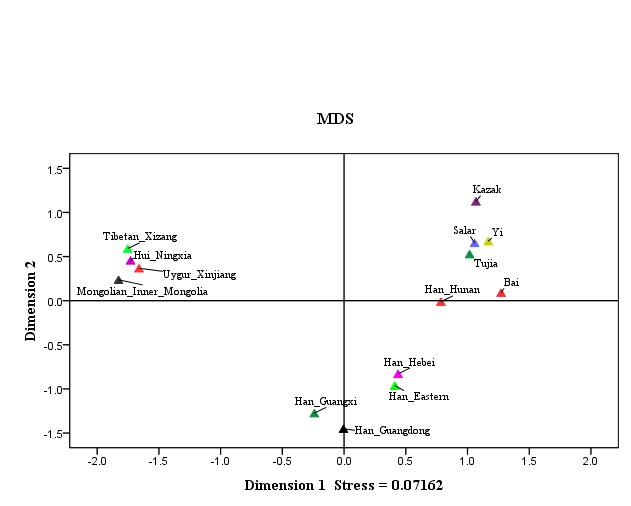

Supplement: Figure_S3_owaf009 [file figure_s3_owaf009.jpeg]

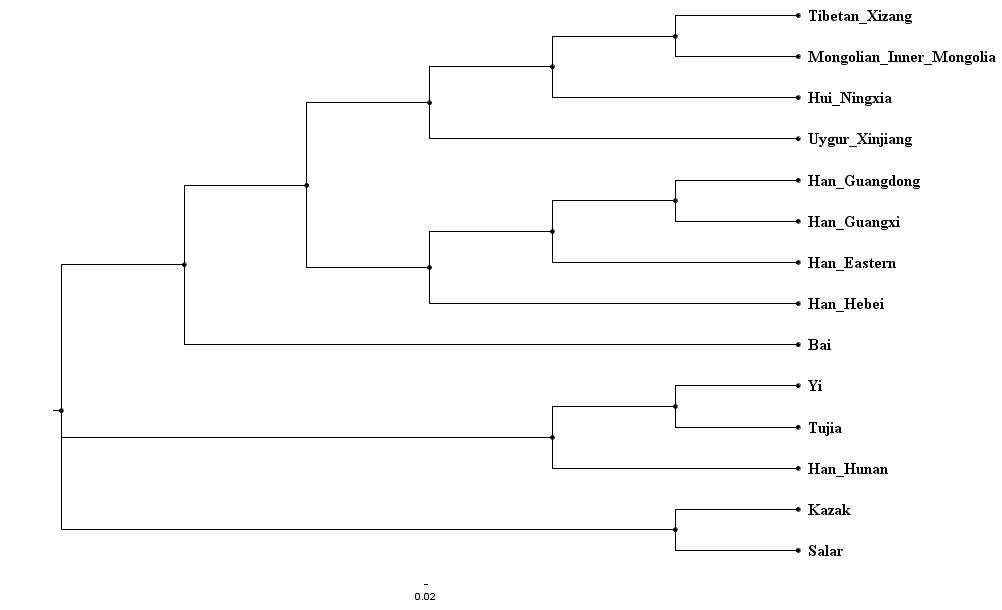

Supplement: Figure_S4_owaf009 [file figure_s4_owaf009.jpeg]
