# Supplementary material for: Correction to: Genetic Polymorphisms of 21 STR Loci of GoldeneyeTM DNA ID 22NC Kit in Five Ethnic Groups of China
Source: Forensic Sci Res. 2025 Jun 4;10(2):owaf009. doi: 10.1093/fsr/owaf009 (PMC12133678; doi:10.1093/fsr/owaf009)
Supplement: Table_S1_owaf009 [file table_s1_owaf009.doc]

**Table S1 Detail information of nine referenced ethnic groups**

| **Population** | **Sample Size** | **Residence** | **Reference** |
| --- | --- | --- | --- |
| Han_Guangxi | 152 | Guangxi, China | [6] |
| Han_Guangdong | 5234 | Guangdong, China | [7] |
| Han_Hebei | 1027 | Hebei, China | [8] |
| Han_Hunan | 501 | Hunan, China | [9] |
| Tujia | 107 | Enshi Tujia Autonomous Prefecture, Hubei, China | [10] |
| Bai | 1158 | Dali, China | [11] |
| Yi | 110 | Yunnan, China | [12] |
| Salar | 120 | Xunhua Salar Autonomous Prefecture, Qinghai, China | [13] |
| Kazak | 114 | Ili Kazak Autonomous Prefecture, Xinjiang, China | [14] |
